# Development of a Primary Human Co-Culture Model of Inflamed Airway Mucosa

**DOI:** 10.1038/s41598-017-08567-w

**Published:** 2017-08-15

**Authors:** Lael M. Yonker, Hongmei Mou, Kengyeh K. Chu, Michael A. Pazos, Huimin Leung, Dongyao Cui, Jinhyeob Ryu, Rhianna M. Hibbler, Alexander D. Eaton, Tim N. Ford, J. R. Falck, T. Bernard Kinane, Guillermo J. Tearney, Jayaraj Rajagopal, Bryan P. Hurley

**Affiliations:** 1 Department of Pediatrics, Harvard Medical School, Boston, MA USA; 2 Department of Pathology, Harvard Medical School, Boston, MA USA; 3 Department of Medicine, Harvard Medical School, Boston, MA USA; 4 Mucosal Immunology and Biology Research Center, Massachusetts General Hospital, Boston, MA USA; 5 Center for Regenerative Medicine, Massachusetts General Hospital, Boston, MA USA; 6 Wellman Center for Photomedicine, Massachusetts General Hospital, Boston, MA USA; 7 Departments of Biochemistry and Pharmacology, University of Texas Southwestern Medical Center, Dallas, TX USA

## Abstract

Neutrophil breach of the mucosal surface is a common pathological consequence of infection. We present an advanced co-culture model to explore neutrophil transepithelial migration utilizing airway mucosal barriers differentiated from primary human airway basal cells and examined by advanced imaging. Human airway basal cells were differentiated and cultured at air-liquid interface (ALI) on the underside of 3 µm pore-sized transwells, compatible with the study of transmigrating neutrophils. Inverted ALIs exhibit beating cilia and mucus production, consistent with conventional ALIs, as visualized by micro-optical coherence tomography (µOCT). µOCT is a recently developed imaging modality with the capacity for real time two- and three-dimensional analysis of cellular events in marked detail, including neutrophil transmigratory dynamics. Further, the newly devised and imaged primary co-culture model recapitulates key molecular mechanisms that underlie bacteria-induced neutrophil transepithelial migration previously characterized using cell line-based models. Neutrophils respond to imposed chemotactic gradients, and migrate in response to *Pseudomonas aeruginosa* infection of primary ALI barriers through a hepoxilin A3-directed mechanism. This primary cell-based co-culture system combined with µOCT imaging offers significant opportunity to probe, in great detail, micro-anatomical and mechanistic features of bacteria-induced neutrophil transepithelial migration and other important immunological and physiological processes at the mucosal surface.

## Introduction

Pathogens accessing the airway mucosa induce inflammation, often leading to bronchitis or pneumonia^1, 2^. Polymorphonuclear neutrophils are among the first responders; neutrophils extravasate from the blood following carefully orchestrated cytokine deployment and adhesion interactions^3–6^ to cross the endothelium^4^. They then traverse the interstitium guided by pericytes, fibroblasts^4^, and collagen breakdown products^7^, and cross the epithelium to reach the site of infection. In certain diseases, neutrophilic recruitment is maladapted. For example, cystic fibrosis, a disease defined by mutations in the cystic fibrosis transmembrane conductance regulator (CFTR) gene, is characterized by chronic airway infection and inflammation. Inflammation results in excessive neutrophil influx, thereby perpetuating pathology^8–10^, ultimately leading to respiratory failure. A comprehensive assessment of neutrophil-epithelial signaling is needed for a better understanding of disease.

A co-culture model has been developed to study neutrophil migration across the airway epithelial barrier^11^. Current understandings of neutrophil transepithelial migration signaling pathways are based almost exclusively on immortalized human lung epithelial cell lines. These cells polarize and form functional barriers, however, they do not feature many physiologically relevant characteristics, such as mucus or beating cilia. Further, these transformed cells may exhibit aberrant signaling pathways compared to primary airway epithelial cells. Our understanding of neutrophil transepithelial migration would benefit by integrating and evaluating physiologically relevant primary airway epithelium that display multiple epithelial cell subtypes as a component of the co-culture model system^12^.

Air-liquid interface (ALI) culturing promotes the generation of pseudostratified mucociliary airway epithelium from airway basal cells cultured on porous transwell filters^13, 14^. Although this platform has been widely used for a variety of studies, it has been hampered by a lack of readily available, substantial quantities of human airway basal cells. Historically, primary airway basal cells are unable to undergo prolonged expansion and their differentiation potential declines with each passage^15^. Recently, we developed a well-defined culture system that allows prolonged expansion of airway basal cells using dual SMAD signaling inhibition, restricting transcription factors that signal TGF-β pathways^16^. Importantly, the expanded airway basal cells retain differentiation potential, exhibit functional airway physiology, and respond appropriately to clinically relevant pharmacologic agents^16^. This new system generates unlimited physiologically relevant human tracheobronchial epithelium for *in vitro* evaluation. In this study, expanded human airway basal cells differentiate on inverted 3 µm pore-sized transwells while maintaining their ability to organize into mucus-producing and ciliated cells. This development allows for the study of inflammatory cell transit using primary airway epithelium. This physiologically relevant platform supports effective neutrophil transepithelial migration to apically-directed exogenous chemoattractants and in response to epithelial infection with *Pseudomonas aeruginosa*. Further, a high-resolution form of optical coherence tomography (termed micro-OCT, μOCT) captured and detailed previously unappreciated cellular dynamics that govern neutrophil transmigratory behavior through airway epithelium^17^. The novel features of this model system include the use of expandable human basal cell-derived epithelium to study neutrophil transmigration, generating a primary co-culture model that has not previously been described, combined with advanced cellular imaging, which offers a physiologically relevant platform to explore micro-anatomical processes at the mucosal surface. Combined, this advanced system represents a significant improvement over traditional human *in vitro* models.

## Results

### Human airway basal cells cultured at an inverted air-liquid interface are able to maintain polarity and functional airway-specific micro-anatomy

**Figure 1:**
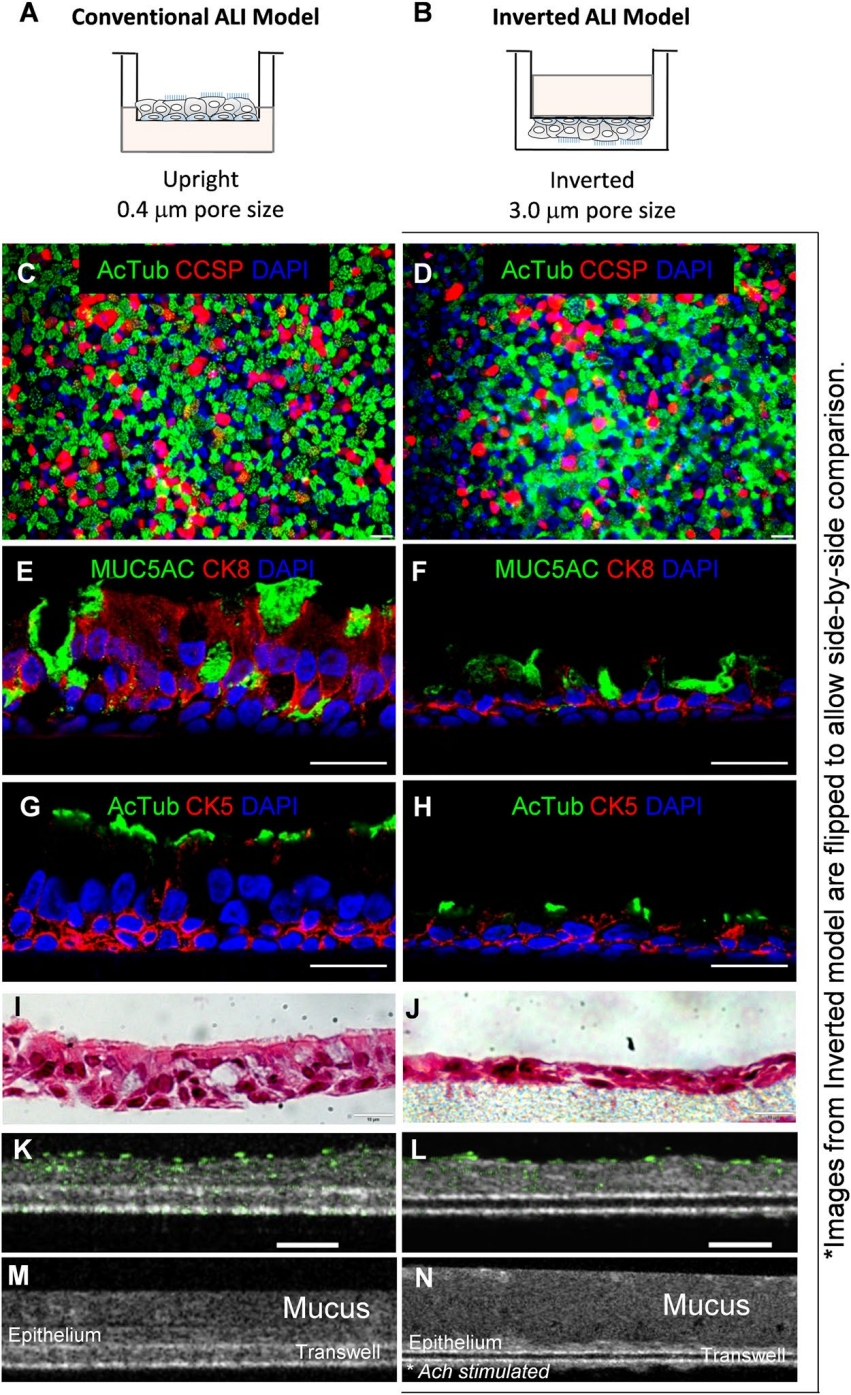
Establishment and characterization of inverted air-liquid interface (ALI) culture system compatible with the study of neutrophil transepithelial migration. In the conventional ALI model (**A**), human airway basal cells are seeded on the top of 0.4 µm pore sized transwell membrane. The medium is added in the lower chamber to initiate and maintain air-liquid interface for the mucociliary differentiation. In inverted ALI model (**B**) for neutrophil migration study, human airway basal cells are seeded on the reverse side of 3 µm pore sized transwell membrane. The medium is added to the upper chamber to initiate and maintain air-liquid interface for the mucociliary differentiation (see Method Section for the detailed description). For comparative purposes, images are oriented with the epithelial layer above the plane of the transwell filter. Conventional (**C**) and inverted (**D**) ALI transwells were wholemount stained for AcTub+ ciliated cells, CCSP+ Club cells, and DAPI+ nuclei. Scale bar, 25 µm. Conventional (**E**,**G**) and inverted (**F**,**H**) transwells were immunostained for differentiation marker CK8, goblet cell marker MUC5AC, ciliated cells marker acetylated tubulin (AcTub) and stem cell marker CK5 in transverse sections of ALI transwell membranes. Scale bar, 20 µm. H&E staining also was used to display pseudostratified epithelium in the conventional (**I**) and inverted (**J**) models. µOCT images of washed human airway basal cell-derived epithelium in conventional (**K**) and inverted (**L**) orientations with a thresholded map of intensity variation over time overlaid in green to accentuate beating cilia, scale bar 50 µm. µOCT analysis of an unwashed human airway basal cell-derived epithelium with mucus intact in conventional (**M**) and inverted (**N**) orientations. Images were analyzed at 3 different areas in >3 separate experiments. Representative images are displayed.

The conventional ALI model, with human airway basal cells seeded on the inner well of a 0.4 µm pore-sized transwell filter, is incompatible with the study of inflammatory transepithelial migration. In order to appropriately integrate ALI culturing with directionally relevant neutrophil transepithelial migration, we modified the conventional ALI by seeding the human airway basal cells on the underside of the transwell. Additionally, 3 µm pore size filters were employed in place of 0.4 µm to enable neutrophil passage (Fig. 1A and B). To minimize the extent to which basal cells pass through 3 µm pores during seeding and differentiation, both faces of the transwell membrane were coated with extracellular matrix. The underside, where human airway basal cells were applied, was coated with 804 G conditioned medium containing laminin-enriched matrix to improve human airway basal cell attachment and maximize differentiation efficiency^16^. The opposite side was coated with 804 G conditioned medium + 5% Matrigel to reduce non-directional human airway basal cell migration through pores and prevent differentiation on the unintended side of the transwell. These modifications were implemented to establish a well-differentiated, polarized ALI airway epithelial layer compatible with neutrophil transepithelial migration co-culture experimental systems.

Both the conventional ALI model (upright, 0.4 µm pore) and the inverted ALI model (inverted, 3 µm pore) support differentiation of human airway basal cells into a physiologic mucosal barrier with mucocilary features on the apical surface two weeks after initiation of ALI culturing conditions (Fig. 1). Immunofluorescent staining reveals airway epithelial components, including ciliated cells (acetylated tubulin^+^) and club secretory cells (CCSP^+^) in both models (Fig. 1C and D). Cross-sectional images also reveal mucociliary differentiation with mucin-secreting goblet cells (MUC5AC^+^) abutted by epithelial cells (CK8^+^) (Fig. 1E and F), and ciliated cells (AcTub^+^) with basal stem cells (CK5^+^) (Fig. 1G and H).

A notable distinction between the conventional and inverted ALI is that the epithelia grown on the inverted 3 µm pore filters are thinner (Fig. 1E–H), as clearly visualized by cross-sectional H&E staining (Fig. 1I vs. J). This difference is not due to inverted growth, as basal cells grown on inverted 0.4 µm pore filters are indistinguishable from those grown in conventional fashion (Supplemental Fig. 1). Rather, when using the larger pore size, some basal cells pass through the 3 µm pores to the opposite face of the filter despite the application of Matrigel to restrict this passage. The phenomenon of sporadic cells crossing, as demonstrated by DAPI (blue, fluorescent stain binding nuclear DNA) staining, appears to reduce the height of the basal cell layer resulting in a thinner polarized airway on the seeding face of the transwell (Supplemental Fig. 2). However, basal cells that managed to migrate to the opposite surface of the transwell did not differentiate into either ciliated or goblet cells, nor did they compromise polarity development of the airway epithelium growing on the seeding face (Fig. 1, Supplemental Fig. 2).

µOCT imaging was used to visualize conventional (0.4 µm) and inverted (3 µm) ALIs. This technique allows for label-free (no fixation, staining, or fluorescent tagging) observation of cellular details at high resolution. Epithelial layers are clearly visualized on both conventional and inverted ALI cultures (Fig. 1K–N). For comparative purposes, images are oriented with the epithelial layer above the plane of the transwell filter. Immunofluorescent staining demonstrated expression of ciliated cell markers in both ALI cultures (Fig. 1C,D,G,H). Video of µOCT imaging confirmed functional beating cilia in inverted ALI (Supplemental Video 1). Measurement of the standard deviations of µOCT pixel intensity in each respective image position over time is displayed in green and further quantifies cilia movement in both conventional (Fig. 1K) and inverted ALI cultures (Fig. 1L). Although epithelium grown in the inverted model has reduced quantity of cilia seen by immunofluorescent (Fig. 1H) and H&E staining (Fig. 1J), the cilia is comparatively functional to that in conventional ALI models, as represented by µOCT analysis of cilia movement.

ALI cultures in both the conventional and inverted models express goblet cell marker MUC5AC (Fig. 1E,F) and exhibit the capacity to produce mucus, which can be observed as a hypo-dense layer above the more opaque epithelial layer (Fig. 1M and N). Mucus is actively produced, and gentle washing of the apical surface with HBSS results in rapid expansion of the mucus layer (Supplemental Fig. 3). Conventional ALIs continuously release mucus at the apical surface, which pools as a compact layer above the epithelium (Fig. 1M). This mucus layer can be removed by additional washing of the epithelial layer with HBSS^+^. The inverted model is less capable of pooling mucus on the apical surface likely due to gravitational forces acting upon the mucus layer. To capture an image of mucus from the apical side of the inverted ALI cultures, epithelial cells were washed and the basolateral surface was treated with 100 µM acetylcholine for one hour prior to imaging by µOCT. The mucus layer on the apical surface appears to be less dense and stretched out, most likely due to the dynamic balance of mucus production, water content and the gravitational pull on the inverted transwell model (Fig. 1N). The scale of Fig. 1M and N are not matched in order to optimize visualization of the mucus layer for each ALI model orientation.

Mucus production and functional cilia are features not commonly observed in polarized airway epithelial cell line cultures, whether grown conventionally or inverted. Certain polarized lung epithelial cell lines develop transepithelial electrical resistance (TEER), as do primary ALI cultures^18^, although TEER can display some variability^19^. The inverted ALI cultures were assessed for the ability to develop measurable TEER. Inverted ALI cultures are capable of developing TEER (median 546 ohms*cm^2^, range 190–1309 ohms*cm^2^, n = 29) for transwells assessed on day 14–29 of ALI differentiation. Although the inverted model displays slightly larger range and lower median value as compared to conventional, upright 0.4 µm pore-sized transwells (median 1508 ohms*cm^2^, range 604–1888 ohms*cm^2^, n = 5), the development of TEER in inverted ALI cultures suggests both functional polarity and barrier integrity similar to what is achieved in the conventional ALI cultured models.

Collectively, these data indicate that human airway basal cells seeded on inverted 3 µm transwells and cultured at an air-liquid interface develop key, micro-anatomical aspects of the airway previously characterized in the conventional human airway basal cell-derived ALI culturing model. Aspects include appropriate polarity, beating cilia, mucus secretion, and development of TEER. Although the inverted ALI culture system appears to develop as a thinner epithelial layer compared with the conventional ALI, it uniquely possesses additional features necessary to establish a co-culture model to examine neutrophil transepithelial migration (grown in an inverted orientation on a transwell filter with 3 µm pores).

### Neutrophils migrate through Human Airway Basal Cell-derived ALI barriers in response to an imposed chemoattractant gradient

**Figure 2:**
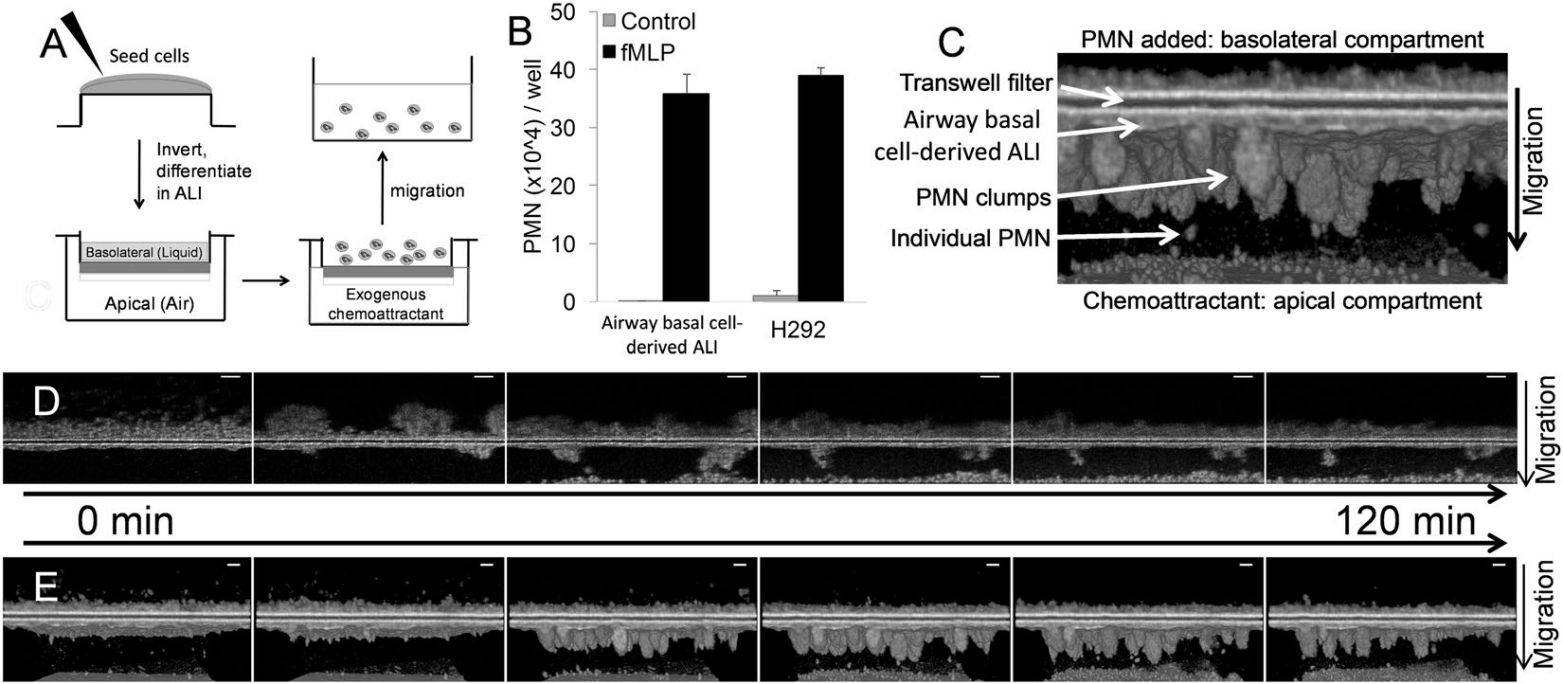
Neutrophil migration in response to an imposed chemoattractant gradient across human airway basal cell-derived ALI cultured epithelial barriers. (**A**) Model schematic of neutrophil transepithelial migration is shown. (**B**) Quantitative MPO read out of fMLP-induced neutrophil migration across epithelium derived from human airway basal cells grown under ALI conditions and H292 cell monolayers, performed in triplicate and replicated ≥3 times with similar results. (**C**) µOCT image of fMLP-induced neutrophil migration is labeled to identify key features within the image. (**D**) Progressive time-lapse µOCT images are shown of neutrophil migration across human airway basal cell-derived epithelial barriers in response to fMLP gradients over a two hour time course in both 2- and (**E**) 3-dimensions. (PMN = neutrophil). Each depicted migration image was examined by µOCT and is representative of 3 separate experiments.

To assess whether neutrophils were capable of migrating across airway epithelial barriers derived from human airway basal cells using the inverted ALI culture system, neutrophils were placed in the basolateral compartment of an inverted ALI transwell. The neutrophil chemoattractant fMLP (100 nM) was placed in the apical compartment to establish a directional concentration gradient (Fig. 2A). Neutrophil transepithelial migration was quantitatively assessed following a two-hour incubation by lysing migrated neutrophils found in the apical compartment and measuring myeloperoxidase (MPO) activity following a two-hour incubation. fMLP gradients produced robust neutrophil migration across inverted ALI cultures. Neutrophil transepithelial migration studies are traditionally conducted by using lung epithelial cell lines to produce inverted polarized monolayers that serve to model the airway mucosa^11^. Therefore, we compared neutrophil migration across the H292 pulmonary epithelial cell line-derived monolayer with migration across human airway basal cell-derived ALI cultures and found that there was no difference in magnitude of neutrophil migration between these two distinct airway epithelial layers (Fig. 2B).

Imaging of neutrophil migration across inverted ALI cultures by µOCT revealed robust migration consistent with quantitative MPO analysis. Furthermore, direct visual µOCT analysis unveiled a novel qualitative perspective of neutrophil transmigratory behavior across the airway. Neutrophils penetrated the epithelium as aggregates in a coordinated manner during the two-hour incubation in the presence of an fMLP gradient (Fig. 2C). Neutrophil migration across inverted ALI cultures was visually captured progressing over time in 2-dimensions (Fig. 2D) and these images were then stacked, permitting 3-dimensional visualization (Fig. 2E). In 2-dimensional µOCT imaging, neutrophils are first seen clustering on the basolateral membrane followed by progressive aggregate movement through the epithelial layer. After breaching the epithelium, neutrophil clusters attached to the apical surface of the epithelium begin to shed individual neutrophils (Supplemental Video 2). In the 3-dimensional µOCT imaging, numerous neutrophil clusters are observed breaching the epithelial barrier then emerging at the apical epithelial surface and shedding neutrophils over the two-hour incubation (Supplemental Video 3).

### Neutrophils migrate through Human Airway Basal Cell-derived ALI barriers following apical infection with Pseudomonas aeruginosa

**Figure 3:**
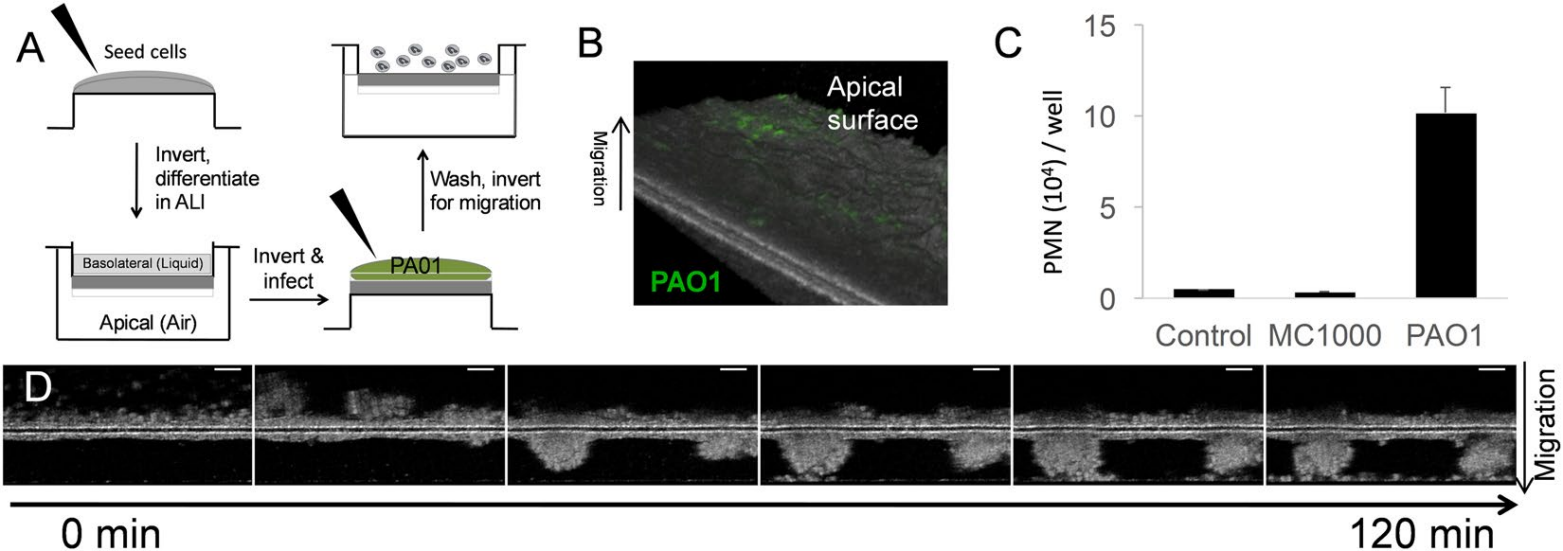
Infection with *Pseudomonas aeruginosa* induces neutrophil migration across the human airway basal cell-derived epithelium. (**A**) Schematic of neutrophil migration induced by PAO1 infection is shown. (**B**) GFP-expressing PAO1 was visualized on mucosal layer of human airway basal cell-derived epithelium using fluorescence-enhanced µOCT imaging. (**C**) Neutrophil migration across human airway basal cell-derived epithelium was assessed by MPO activity in response to epithelial infection with PAO1 and a non-pathogenic *E.coli*, MC1000, performed in triplicate and replicated >3 times with similar results. (**D**) Progressive time-lapse µOCT images are shown of neutrophil migration across the human airway basal cell-derived epithelium in response to apical epithelial PAO1 infection. µOCT images are representative of 3 separate experiments.

To test whether infection of inverted ALI cultures on the apical surface would trigger neutrophil transepithelial migration, we infected the epithelium for one hour with *Pseudomonas aeruginosa* (PAO1) followed by a washing step prior to basolateral application of neutrophils (Fig. 3A). GFP-expressing PAO1 could be observed associating with ALI cultures, demonstrating interaction between bacteria and epithelial cells (Fig. 3B). Quantitative assessment of the magnitude of PAO1-induced neutrophil migration was determined by MPO activity in the apical well. Infection with PAO1 resulted in a significant increase in the numbers of neutrophils migrating when compared to infection with an equal amount of non-pathogenic *E.coli* (MC1000) or mock infection (buffer) control (Fig. 3C). Neutrophil migration across the human airway basal cell-derived ALI cultures were again visualized by µOCT over time (Fig. 3D). When visualized in 2-dimensions, neutrophils were seen clustering on the basolateral surface, then breaching the epithelium, generating tightly associated neutrophil aggregates on the apical surface of the epithelium before neutrophils individually detach (Supplemental Video 4). Further, we were able to observe qualitative differences in neutrophil migration dynamics when comparing migration to fMLP gradient and migration to *P. aeruginosa* infection. On the basolateral surface, neutrophils undergo similar patterns of clumping prior to epithelial breach in both infection-mediated and fMLP-mediated chemotaxis. Neutrophils migrating towards fMLP cross the epithelium and appear to form smaller clusters on the apical surface then detach from the cluster more readily, while neutrophils migrating in response to PAO1-induced epithelial signaling appeared to form larger, more cohesive masses on the apical surface (Supplemental Video 2: fMLP-induced migration, Supplemental Video 4: PAO1-induced migration).

### Interference with hepoxilin A3 (HxA3) reduces *P. aeruginosa*-induced neutrophil migration across human airway basal cell-derived ALI barriers

**Figure 4:**
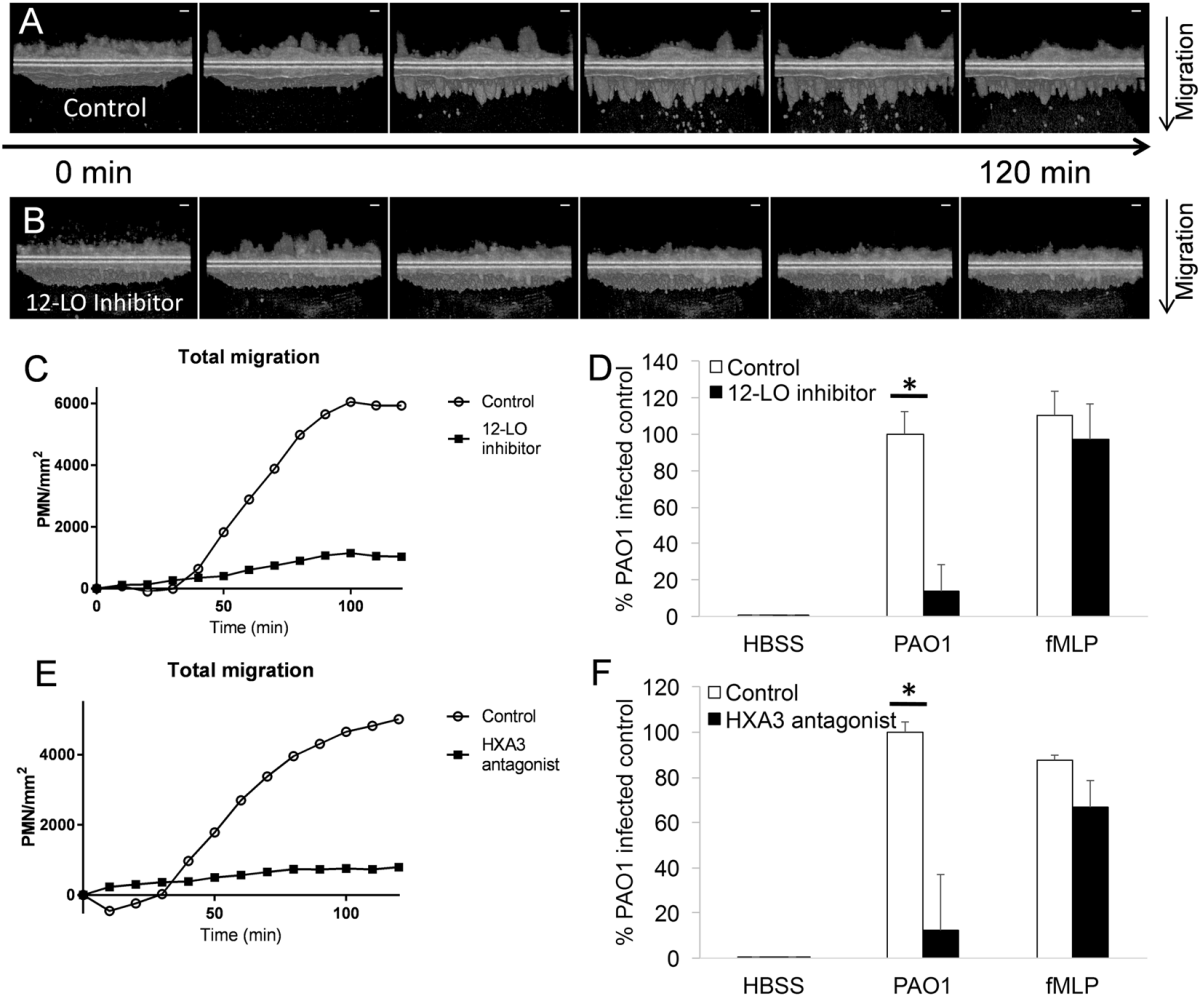
Inhibition of hepoxilin A3 reduces PAO1-induced neutrophil migration across human airway basal cell-derived epithelial barriers. Time-lapse µOCT images of neutrophil transepithelial migration following epithelial treatment with (**A**) vehicle control (DMSO 1:1000) or (**B**) CDC, a 12-LO inhibitor. (**C**) Neutrophil migration of single, representative experiment was quantified by µOCT time course plot following epithelial treatment with 12-LO inhibitor CDC, or vehicle control (DMSO 1:1000). (**D**) Neutrophil migration was quantified using myeloperoxidase in response to PAO1 infection or an imposed gradient of fMLP following epithelial treatment with 12-LO inhibitor CDC, or vehicle control (DMSO 1:1000). Data displayed combined results from two internally controlled, independent experiments (PAO1 infection: n = 4 for each condition). (**E**) Time course plot comparison of single, representative µOCT imaging quantifies migration visualized following epithelial treatment with HxA3 antagonist or control (HBSS) at the apical surface. (**F**) Neutrophil migration in response to epithelial infection with PAO1 or an imposed gradient of fMLP in the presence or absence of HxA3 antagonist was quantified by myeloperoxidase assay. Data combined results from two internally controlled, independent experiments (PAO1 infection: n = 4 for each condition). µOCT images are representative of at least 2 separate experiments.

HxA3 plays a critical role in infection-induced transepithelial migration based on data generated from the traditional epithelial cell line-derived co-culture model and from *in vivo* experiments^20–23^. Epithelial cells produce the lipid neutrophil chemotactic agent, HxA3, in response to infection and this signal is responsible for driving neutrophils across the epithelium from the basolateral to the apical surface^21^. 12-Lipoxygenase (12-LO) converts arachidonic acid into HxA3 and inhibition of this enzyme significantly impacts infection-induced neutrophil recruitment^20^. Of note, neutrophil chemoattractants, such as fMLP, do not rely on infection-induced epithelial release of HxA3 to stimulate migration when applied directly to the apical side creating a chemotactic gradient^20, 21^. To determine whether interfering with the ability of inverted ALI cultures to synthesize HxA3 impacted *P*. *aeruginosa*-induced neutrophil transepithelial migration, we pre-treated ALI cultures derived from human airway basal cells with CDC, an inhibitor of 12-LO. Pre-treatment of epithelia with vehicle control did not impact the ability of PAO1 to induce robust neutrophil transepithelial migration (Fig. 4A, Supplemental Video 5). CDC pre-treatment of the epithelia, however, resulted in a notable decrease of neutrophil transepithelial migration relative to vehicle control, as observed by µOCT (Fig. 4B, Supplemental Video 6). In the setting of both CDC pre-treatment and vehicle control pre-treatment of the infected epithelium, neutrophils were observed undergoing activation on the basolateral surface of the epithelium as they form heaping clumps of cells (Supplemental Videos 5 and 6). However, in the context of epithelial pre-treatment with the 12-LO inhibitor (CDC), very few neutrophils proceed to penetrate the infected epithelial layer and reach the apical side (Supplemental Video 6). Quantification of µOCT imaging using a computer algorithm reveals minimal transepithelial migration of neutrophils in the setting of ALI cultures pre-treated with CDC, whereas over 6000 neutrophil/mm^2^ transmigrated when the ALI cultures were pre-treated with vehicle control (Fig. 4C). In a separate quantitative analysis by MPO activity, CDC pre-treatment significantly decreased neutrophil migration compared with vehicle control (p < 0.05). Epithelial treatment with CDC did not affect neutrophil transepithelial migration in response to an imposed gradient of fMLP (Fig. 4D), as fMLP gradient mediated chemotaxis does not rely on epithelial 12-LO-mediated HxA3 release to drive neutrophil transmigration. Thus, PAO1-induced neutrophil migration across human airway basal-derived ALI cultures specifically involves 12-LO activity.

We then tested whether antagonism of HxA3 function would affect neutrophil transepithelial migration in the human airway basal cell-derived inverted ALI culture system. Neutrophil migration across PAO1-infected human airway basal cell-derived cultures was assessed in the presence or absence of an HxA3 antagonist applied to the apical side. The HxA3 antagonist employed is a structural analogue of HxA3 that has been previously demonstrated to interfere with PAO1-induced neutrophil migration across H292 airway epithelium^22^. Quantitative analysis of µOCT images revealed PAO1-infected human airway basal cell cultures did not feature much neutrophil transepithelial migration when HxA3 antagonist was present, which starkly contrasts with the observed robust PAO1-induced transepithelial migration in the absence of antagonist (Fig. 4E). The appearance of negative migration in the control case is an artifact of the analysis algorithm, which detects small volumes of settling debris near the epithelium, elevating the baseline reading and thus resulting in a negative differential count of bright image voxels at time points after the debris settled and before neutrophils began migrating. Quantitative read-out of MPO activity in the apical well confirmed a significant decrease of migration in PAO1-induced migration in the setting of HxA3 antagonist (p < 0.05), while migration to an imposed gradient of an unrelated chemoattractant (fMLP) was not affected (Fig. 4F).

## Discussion

ALI culturing is a well-established method to generate functional mucociliary epithelial barriers from mouse and human airway basal cells^13, 14^. Such *in vitro*-differentiated models exhibit multi-cellular diversity and physiologic functioning that resembles the airway surface *in vivo* ^13, 24–28^. The ability to expand and differentiate human airway basal cells into matured airway epithelium provides a powerful *in vitro* platform to study a myriad of human respiratory diseases, including cystic fibrosis^[15,16, 27^.

A novel model to explore neutrophil breach of the airway mucosa has been developed herein using exclusively primary cells derived from a donor without lung disease. Advances in basal/stem cell culturing techniques were harnessed to create reproducible, physiologically relevant air-liquid interface airway mucosal barriers. These airway mucosal barriers feature inverted polarity and are cultivated upon a transwell filter with a 3 µm sized pore, enabling the investigation of neutrophil transepithelial migration and underlying molecular mechanisms thereof. Further, we capitalize on recent advances in live cellular imaging and analysis using µOCT to provide insight into cellular mechanisms associated with neutrophil transepithelial migration. This human airway basal cell-derived ALI model, coupled with µOCT imaging, can be used to test hypotheses generated from studies using cell line-based co-culture systems to expand our understanding of processes that underlie neutrophil transepithelial migration in a context that more closely approximates the setting of the airway surface.

Mechanistic understanding regarding the critical inflammatory processes that orchestrate neutrophil migration across mucosal epithelial barriers, in response to infection or otherwise, has been largely gained through investigations either *in vivo* or with *in vitro* co-culture systems involving immortalized human cell lines^5, 6, 21, 29–33^. Animal models are valuable in the exploration of neutrophil recruitment to the airspace as they provide an appropriate *in vivo* context. However, *in vivo* models lack the precision necessary to specifically interrogate key mechanisms such as transepithelial migration within the overall recruitment process. Additionally, *in vivo* modeling represents a non-human experimental system that introduces important caveats when modeling human disease. *In vitro* co-culture systems represent an excellent platform for deciphering neutrophil transepithelial migration at the mechanistic level. Polarized human epithelial cell lines are readily adaptable to the inverted co-culture model and can provide cellular and molecular mechanistic insight in a highly reproducible system. However, the lung epithelial cell lines commonly employed are immortalized and lack the true micro-anatomical complexity of the airway surface, particularly epithelial cell subtypes that promote mucus secretion and ciliogenesis. Therefore, a more robust approach to understanding neutrophil breach of the airway mucosa would incorporate primary human epithelial cells into these co-culture models to better approximate the human airway surface.

The use of primary epithelia in neutrophil transepithelial migration co-culture models has not been widely adopted, partly because of the high costs and technical challenges associated with isolation, expansion, and differentiation of primary airway cells *in vitro*. A recently developed feeder-free universal culture system allows unprecedented expansion of multiple epithelial progenitor cells, including human airway basal cells. After expansion, the airway basal cells maintain their morphology, stem cell markers, and differentiation potential^16^. Further, human airway basal cells cultured on transwells under ALI conditions develop into a mature airway mucosa. Methodological adaptations to conventional ALI models were necessary to establish an inverted orientation on a transwell with larger pores to permit neutrophil passage (3 µm vs. 0.4 µm). It is important to note that this experimental modification did result in the development of a more compressed epithelium that we attributed to the cultivation on 3 µm pore sized transwell filters rather than the inverted orientation. This thinner epithelium may influence some aspects of the molecular and cellular mechanisms to be explored in future applications of this model system and should be considered when interpreting results and when relating to processes that occur *in vivo* in the context of human disease. However, despite this caveat, integration of the primary ALI airway mucosa model into the neutrophil transepithelial experimental co-culture system offers a more physiologic assessment of this important inflammatory process over currently available alternative approaches. It also enables more targeted analysis of host-pathogen interactions whereby the applied pathogen is encountering a physiologically relevant primary airway mucosa with multiple airway epithelial subtypes rather than a monoculture of transformed epithelial cells.

The human lung basal cell-derived airway epithelium cultured to be compatible with neutrophil transepithelial migration studies recapitulates multiple salient features of human airway. Mucus production and beating cilia contribute an additional layer of physiologic complexity that can be leveraged towards the study of neutrophil trafficking. Specifically, this human primary epithelial model incorporates appropriate cellular architecture, comprised of pseudostratified epithelium consisting of basal stem cells as well as distinct and apically oriented ciliated, secretory, and mucin-secreting goblet cells. These features may now be considered through careful study design to determine their potential impact on bacteria-induced neutrophil transepithelial migration. Conversely, the inclusion of migrating neutrophils could also inform mechanisms underlying mucus and cilia function. Longer-term infections, biofilms, or interaction with neutrophil byproducts, such as neutrophil extracellular traps (NETs) in association with ALI models can also be integrated into the model system.

Beyond establishing this advanced, physiologically relevant co-culture system modeling neutrophil breach of mucosal barriers, we acquired dynamic, in-motion perspectives of the neutrophil transmigratory process at 1 µm resolution using µOCT imaging^17, 34^. μOCT is a high-resolution implementation of optical coherence tomography (OCT), which uses optical interference with a reference beam to determine the depths of back-scattered light generated by a sample, creating a cross-sectional map of optical reflectance. Imaging using µOCT does not require sample labeling or manipulation as the index of refraction differences within cells and tissue yield high natural contrast. This natural contrast allows μOCT to reveal live-action, detailed, three-dimensional cellular processes manifesting over time. This technique has been successfully used to visually characterize airway functional microanatomy, including cilia beat frequency and mucus layer depth^34, 35^. µOCT pairs image acquisition with multiplexed quantification of previously inaccessible micro-physiological processes for study following exposure to relevant stimuli. In this study, µOCT allowed characterization of mucus production generated by the ALI and cilia beat activity, in addition to visualization of neutrophil-epithelial interactions and migration following chemoattractant signaling within the airway. The effects of active mucus generation and beating cilia on neutrophil-epithelial crosstalk, as well as the dynamic effects of migrating neutrophils on epithelial integrity, are features that warrant further detailed study. In addition, future studies geared toward applying μOCT imaging *in vivo* would enable visualization and characterization of neutrophil breach of the mucosa in true physiological context of the airway and would ultimately reveal whether cellular mechanisms underpinning neutrophil transepithelial migration described herein are representative of what occurs in the infected and inflamed lung.

*In vitro* co-culture models involving human airway basal cell-derived airway epithelial layers reproducibly and reliably facilitate neutrophil migration in response to chemotactic gradients generated by epithelial infection with *P. aeruginosa* (PAO1)^20^. Cell line-based transepithelial migration models have shown that *P. aeruginosa* infection induces epithelial production of HxA3, a 12-LO metabolite of arachidonic acid, which draws neutrophils into the apical compartment^20–22^. In this study, we have confirmed that human airway basal cell-derived airway epithelial layers rely on similar mechanisms. PAO1-induced transepithelial migration was disrupted by 12-LO inhibitors and was blocked following administration of HxA3 structural antagonists, as was previously reported using cell line-based co-culture models. These results support the concept that interfering with epithelial-produced HxA3 may serve to dampen infection-induced inflammatory damage by reducing the number of neutrophils that breach the airway epithelial barrier.

The use of µOCT imaging to visualize neutrophil migration across human basal cell-derived ALIs provides important descriptive information that is not fully appreciated using standard end-point imaging and read-out assays. Specifically, we observed common patterns of neutrophil activation and clumping on the basolateral aspect of the ALI prior to migration towards both an infected epithelium or exogenous fMLP. Signaling mechanisms on the basolateral aspect of the epithelium are likely important to neutrophil influx into the airway and warrant further research. We also discovered that neutrophils that migrate across the epithelium aggregate in unique patterns, depending on the stimulus. Specifically, PAO1-mediated neutrophil migration across a human basal cell-derived epithelium resulted in large, cohesive clumps, while neutrophil migration towards fMLP resulted in smaller clumps with neutrophils that disassociated from the clumps more readily. Better understanding the mechanisms leading to these observed migratory patterns will allow further insight into infection-mediated epithelial signaling pathways, which could have important clinical implications for treatment of inflammatory lung diseases, infectious or otherwise, that involve neutrophil recruitment. Therefore, the inverted (3 µm) ALI co-culture model coupled with µOCT imaging will be useful to further interrogate signaling pathways that orchestrate bacteria-induced neutrophil transepithelial migration.

In summary, we have described an advanced model for studying neutrophil trafficking mechanisms that enhances the existing inverted co-culture models by incorporating human airway basal cells and thereby significantly improving our ability to model human physiologic and innate immune mechanisms in the airway in the context of disease and infection. This system, paired with advanced imaging, creates an unprecedented opportunity to integrate disease-oriented and personalized study of inflammation within the airway. The study herein relied upon basal cells from a single donor without lung disease, however, future investigation can incorporate basal cells from a variety of individuals, including those with genetically defined lung disease, to assess potential genetic differences that may influence development or performance of the co-culture model system. In conclusion, this model provides an opportunity to significantly expand our understanding of neutrophil-epithelial crosstalk in the human airway.

## Materials and Methods

### Growth and maintenance of epithelial cells

The H292 cell line, obtained from American Type Tissue Culture, is a human pulmonary epithelial cell line derived from a patient with mucoid pulmonary carcinoma. H292 cells were maintained in DMEM/F12 (1:1) culture media with 10% heat-inactivated fetal bovine serum plus 100 I.U./mL penicillin and 100ug/mL streptomycin. H292 cells were seeded onto collagen coated inverted transwells with permeable (3 µm pore size) polycarbonate membrane Inserts and a culture area of 0.33 cm^2^ (Corning product #3415). Transwells were incubated overnight to allow cells to attach to the bottom surface of the filter, then reverted into a 24-well tissue culture plate with media placed on both sides of the transwells. H292 monolayers were maintained in culture media for at least seven days to ensure formation of functional barriers prior to use for migration assays^11^.

### Human airway basal cell isolation, culture and mucociliary differentiation on air-liquid interface

Human airway basal cells, for this study, were isolated and expanded from airway tissue harvested from a single donor without lung disease through the New England Organ Bank under an IRB-approved protocol (#2010P001354), as previously described^16^. Informed consent was obtained by New England Organ Bank and provide to investigators and all experiments were performed in accordance with the IRB-approved protocol. In brief, using a previously published basal cell isolation protocol^16^, EpCAM^+^ epithelial basal cells were isolated from trachea and mainstem bronchi then cultured in complete SAGM (Lonza, Cat. CC-3118), with prolonged propagation. Under these culture conditions, staining of EpCAM^+^ epithelial basal cells at passage 1 resulted in 100% positivity for lung transcription factors NKX2.1, SOX2, FOXA2 and general stem cell markers p63 and KRT5, uniquely identifying them as pure adult airway basal cells^16^. For the purposes of consistency between experiments performed within this study, basal cells isolated from a single donor were used between passages 4–8. Basal cells were cultured using the dual SMAD inhibitor protocol^16^, split, and seeded onto transwell membranes with a seeding density of >6000 cells/mm^2^.

In conventional ALI mode, cells were seeded onto the up-facing surface of 6.5mm transwells with Polyester Membrane Inserts with 0.33 cm^2^ culture area and 0.4 µm sized pores (Corning product #3470) pre-coated with 804 G medium. Seeding and growth occurred in standard fashion^16^. The inverted ALI model for study of neutrophil transepithelial migration used the same transwells as the H292 inverted model: Transwells with permeable (3 µm pore size) polycarbonate membrane Inserts and a culture area of 0.33 cm^2^ (Corning product #3415). For seeding of basal cells on 3 µm transwells, a two-step process is required: First, transwells were flipped and 80–100 µl of 804 G conditioned medium was added to the top of the membrane and incubated at 37 °C overnight. The following day, coating medium was removed and replaced with 80 µl of the airway basal cells suspension in SAGM for a four to six hour time frame to allow adhesion. The extra SAGM medium and any unattached cells were then carefully aspirated and transwells were returned to receiving wells containing 500 µl complete SAGM for airway basal cell recovery and expansion. One hundred microliters of 804 G conditioned medium +5% Matrigel (BDBiosciences, 354230) was then added to coat the up-facing surface of the transwell in the upper chamber. After incubation overnight at 37 °C, SAGM medium in the lower chamber and the coating medium in the upper chamber were removed and replaced with complete SAGM. For both conventional and inverted ALI models, the growth medium (complete SAGM) remained in both the upper and lower chamber for 1–2 days to ensure cellular confluence. The media in both chambers was then replaced with complete Pneumacult-ALI medium (StemCell Techonology, Cat. 05001) or Vertex ALI medium^36^ for another day. The next day, ALI medium was added only to the lower chamber (conventional model) or only in the upper chamber (inverted model) to initiate airway-liquid interface (count as day 0). Media was changed every 1–2 days until differentiation was well established. Ciliogenesis was monitored by inverted-phase microscopy.

ALIs used in experiments were cultured for at least 9 days to allow for full maturation of both cilia and goblet cells, but no more than 34 days to avoid overgrowth or loss of epithelial barrier. Transepithelial electrical resistance was assessed prior to migration assays to ensure the establishment of polarized epithelial barrier using a voltmeter (EVOM2, Epithelial Voltohmmeter, World Precision Instruments, Inc.).

### ALI harvest, sectioning, and staining

To stain for differentiation markers, the membranes were fixed with 4% paraformaldehyde (PFA) at room temperature for 10 min, washed, and permeabilized with PBS + 0.2% Triton X-100. The membranes were used for wholemount staining or were embedded in optimal cutting temperature compound for 6 µm frozen/paraffin sections. For immunofluorescence staining, the ALI membranes or ALI sections were incubated with the primary antibodies diluted in PBS + 1% BSA for two hours at room temperature or overnight at 4 °C (>16 hrs). Following incubation, the membranes or slides were rinsed four times with PBS + 0.2% Triton X-100, and incubated with secondary antibodies at room temperature for one to two hours. After incubation, the membranes or slides were rinsed four times with PBS + 0.2% Triton X-100, then stained with DAPI (4’,6-diamidino-2-phenylindole) by adding sufficient 0.1 µg/ml DAPI solution for three minutes before mounting and imaging. Stainings were visualized using an Olympus Fluoview FV10i Confocal Microscope or a Nikon A1 Confocal Laser microscope. The whole-mount staining on ALI transwell membranes was visualized with the Olympus IX81 inverted fluorescence microscope. Images were captured at multiple focal planes and combined using MicroSuite FIVE (Olympus Soft Imaging Solutions) and Extended Focal Imaging (EFI) module to create a single in-focus image, capturing the cellular complexities of the thicker ALI cultures. The primary and secondary antibodies used in this study include acetylated tubulin (Sigma, Mouse monoclonal, T7451, 1:10,000), CCSP (Santa Cruz Biotechnology, Goat polyclonal, sc-9772, 1:200), MUC5AC (Thermo Scientific, Mouse monoclonal, 2013–05, 1:500), CK5 (abcam, rabbit polyclonal, ab53121, 1:500) and CK8 (Developmental Studies Hybridoma Bank, Rat monoclonal, TROMA-I, 1:50). The secondary antibodies conjugated with Alexa Fluor (488 and 594) were purchased from Life Technologies and used at 1:500. For each stained image visualized by microscopy, three different areas of the transwell membrane were analyzed in more than three separate experiments. Representative staining images are displayed.

### Neutrophil isolation

Neutrophils were isolated, using an established technique, from whole blood of healthy volunteers after receipt of written informed consent under an approved Institutional Review Board protocol (#1999P007782) at the Massachusetts General Hospital^11^. All experiments were performed in accordance with the IRB-approved protocol. Briefly, blood was drawn by venipuncture into syringe containing anticoagulant, acid citrate/dextrose, and subjected to centrifugation to remove plasma and mononuclear cells. A 2% gelatin sedimentation technique followed by lysis of red blood cells (RBCs) with NH_4_Cl was then performed. Cells were subsequently washed and resuspended in HBSS without calcium or magnesium (HBSS-) at a concentration of 5 × 10^7^ neutrophils/ml. This neutrophil isolation technique allows for isolation of functionally active neutrophils (>98%) at 90% purity^37^.

### Bacterial strains

*Escherichia coli* (MC1000)^11^ and *Pseudomonas aeruginosa* (PAO1)^11^ were grown aerobically in Luria-Bertani broth overnight at 37 °C in a shaking incubator. Prior to the experiments, each bacterial suspension was resuspended in HBSS and diluted to a concentration of 6 × 10^7^ CFU/ml HBSS. For migration experiments, transwells were infected with 25 µl of 6 × 10^7^ CFU/ml bacteria for 1 hour, then washed. To allow µOCT visualization of GFP-expressing PAO1^38^ adherent to the epithelium, transwells were placed in wells containing 1 ml of GFP-expressing PAO1 6 × 10^7^ CFU/mL HBSS for five hours, then washed immediately prior to imaging^39^.

### Drug treatment

Cinnamyl-3,4-dihydroxy-α-cyanocinnamate (CDC; Enzo Life Sciences), a 12-lipoxygenase inhibitor, was suspended as per manufacturer’s instructions and diluted to 50 µM. Vehicle controls consisted of DMSO diluted with HBSS at a matching dilution factor. The HxA3 (100 µg/ml) antagonist was characterized in previous studies under the name PN-II-218–36 and synthesized in the laboratory of Dr. J.R. Falck at UT Southwestern Medical Center.

### Neutrophil transepithelial migration

Assays were adapted from a neutrophil transepithelial migration co-culture model system^11^. Epithelial layers grown on transwells were washed and equilibrated in HBSS. For experiments involving epithelial pre-treatments, epithelia were incubated in chemical inhibitor (CDC) or vehicle control (1:1000 DMSO) for two hours. Transwells were washed, inverted and the epithelial surface was infected with 25 µl of 6 × 10^7^ CFU/ml bacteria (MC1000 or PAO1) or treated with HBSS for one hour prior to washing. Infected transwells and uninfected controls were then placed in wells containing either HBSS, or, in certain experiments, HxA3 antagonist. Additional uninfected controls were placed in exogenous chemoattractant, fMLP (100 nM, Sigma). Neutrophils, isolated and resuspended to a concentration of 5 × 10^7^ neutrophils/ml, were diluted 1:10 in HBSS + and 200 µl of this dilution were placed in the basolateral compartment for a final number of 1 × 10^6^ neutrophils/24-well transwell. Migration was allowed to progress for two hours while at 37 °C, 5% CO2. After two hours, transwells were discarded and migrated neutrophils were quantified using a peroxidase assay to assess neutrophil myeloperoxidase (MPO) activity^11^. For individual experiments, a standard curve was generated to define number of neutrophils migrated per myeloperoxidase activity (OD at 405) (linear regression, R^2^ of >0.99). When combining data from experiments run on separate occasions, migration was normalized to percent of PAO1-infected controls to allow comparison between groups and across experiments.

### µOCT imaging

High-resolution micro-optical coherence tomography (µOCT) was used to acquire images of neutrophil migration through cell cultures. The instrument was custom-built to provide optical resolutions of 2 µm in the lateral/horizontal direction, and 1 µm in the axial/vertical direction. µOCT imaging methods have been previously reported^17, 34^. Briefly summarized, the µOCT instrument measured the depth-resolved reflectivity of a sample in response to broadband light generated by a supercontinuum laser source (NKT Photonics, Birkerod, Denmark). Motorized mirrors redirected the beam to produce 2D or 3D cross-sectional OCT images. The µOCT instrument was used in an inverted configuration; the imaging laser beam was directed to the sample from below. A custom holder was constructed with a transparent OCT-compatible bottom to hold the transwell containing the epithelium and neutrophils at a distance of approximately 100 µm from the glass bottom.

µOCT neutrophil imaging assays were performed using 3D video imaging. A 3D µOCT volume was acquired every 10 minutes over two hours immediately after the placement of neutrophils into the basolateral compartment. The sample was maintained near 37 °C during migration by incandescent heat source.

To obtain neutrophil migration counts as a function of time, µOCT volume sequences were loaded on the image processing software ImageJ. Regions of interest were selected and cropped. For each time point, the number of voxels exceeding a threshold brightness was counted by ImageJ and divided by the corresponding number of voxels from a single isolated neutrophil to yield the number of neutrophils in the selected region at a single point in time^17^. Results of this analysis were portrayed in time course plot comparison.

Co-registered fluorescence was measured by co-aligning a 488 nm laser into the µOCT infrared beam path^40^. Fluorescence emission was separated from the µOCT beam by a dichroic filter with a cutoff wavelength of 605 nm and an emission filter was selected to maximally reject excitation (488 nm) and stray OCT light (600 + nm). A photodiode detected filtered fluorescence emission, with one measurement made per µOCT image column. Though the fluorescence and µOCT beams are co-registered, the fluorescence emission is not natively depth-resolved. Fluorescence information was combined with µOCT data in volume renderings by modulating the green channel saturation of the bright apical surface of epithelium in the 3D µOCT volume. (Supplemental Fig. 4)

The presence of cilia was analyzed by measuring the standard deviations in µOCT pixel intensity in each respective image position over time. Normalized and thresholded maps of standard deviations were superimposed on time-averaged 2D µOCT cross-sectional images as a green channel overlay.

### Statistics

Data were reported as means ± SD and compared by unpaired, two tail Student’s t-test. A p value ≤ 0.05 was considered significant.

## Electronic supplementary material

Supplementary Information

Supplemental Video 1

Supplemental Video 2

Supplemental Video 3

Supplemental Video 4

Supplemental Video 5

Supplemental Video 6
