# Supplementary material for: Development of a Primary Human Co-Culture Model of Inflamed Airway Mucosa
Source: Sci Rep. 2017 Aug 15;7:8182. doi: 10.1038/s41598-017-08567-w (PMC5557980; doi:10.1038/s41598-017-08567-w)
Supplement: Supplementary file 1 — Supplementary Information [file 41598_2017_8567_MOESM1_ESM.pdf]

Supplementary Data Title Page

Development of a Primary Human Co-Culture Model of Inflamed Airway Mucosa

Lael M. Yonker, MD[1,4]*, Hongmei Mou, PhD[1,4,5]*, Kengyeh K. Chu, PhD[2,6], Michael A. Pazos, PhD[1,4], Huimin Leung, PhD[2,6], Dongyao Cui [2,6], Jinhyeob Ryu[6], Rhianna M. Hibbler[4], Alexander D. Eaton[4], Tim N. Ford[2,6], J. R. Falck, PhD[7], T. Bernard Kinane, MD[1], Guillermo J. Tearney, MD, PhD[2,6], Jayaraj Rajagopal, MD[3,5], Bryan P. Hurley, PhD[1,4]

*co-first authors

[1] Department of Pediatrics, [2] Department of Pathology, [3] Department of Medicine, Harvard Medical School, Boston, MA, USA

[4] Mucosal Immunology and Biology Research Center, [5] Center for Regenerative Medicine, [6] Wellman Center for Photomedicine, Massachusetts General Hospital, Boston, MA, USA

[7] Departments of Biochemistry and Pharmacology, University of Texas Southwestern Medical Center, Dallas, TX

Supplemental Figure 1: Conventional and inverted ALIs grown on 0.4μm pore sized transwells display comparable epithelial thickness and architectural make-up. Human airway basal cells were seeded on either the upright **(A)** or inverted **(B)** surface of 0.4μm pore sized transwell membrane, and visualized by wholemount staining (upper panel) and cross-section staining (lower panel) of CCSP+ club cells, AcTub+ ciliated cells and DAPI+ nuclei on ALI transwell membranes. Scale bar: 20μm.

Supplemental Figure 2: **Undifferentiated basal cells observed on non-seeding face of 3μm transwells.** Expanded human airway basal cells were differentiated on the inverted surface of 0.4μm **(A)** or 3μm **(B)** transwells. **(C)** The epithelium on both sides of the transwells was stained

for AcTub and MUC5AC by immunofluorescence. 3D view of immunofluorescence was generated using Z-stack scanning by confocal microscope and reconstructed by using ImageJ software. Scale bar, 20μm.

Supplemental Figure 3: **Washing the apical ALI surface results in rapid expansion of the mucus layer.** The apical surface of the conventional, upright ALI derived from human airway basal cells was washed with 200μl HBSS x3 then imaged by μOCT.

Supplemental Figure 4: **Schematic diagram of μOCT and fluorescence system.** μOCT components: Supercontinuum laser (SCL) power was directed towards sample by collimating and focusing lenses (L) through single mode fiber (SMF), which contained common-path interferometer optics. Returning light was directed by the beam-splitter (BS) towards a diffraction grating (G). The spectrally dispersed light was then focused onto a line scan camera (LSC), which output raw spectrograms through a CameraLink (CL) interface to an image acquisition board (IMAQ) installed in a PC. The PC also controlled scanning by way of a data acquisition card (DAQ), which controlled a piezoelectric motor (PZM) using an analog output (AO). Fluorescence components: Light from a 488 nm laser was co-aligned with μOCT beams using a dichroic mirror (DM). Fluorescence emission light was separated from excitation by another DM, and filtered by an emission filter (EM) prior to detection by an avalanche photodiode (APD). The signal from the APD was digitized by the DAQ.

Video 1: Two-dimensional video μOCT imaging reveals beating cilia on inverted (3μm) ALI.

Video 2: Two-dimensional μOCT video imaging is used to visualize neutrophil migration across human airway basal cell-derived epithelial layer towards the exogenous chemoattractant, fMLP.

Video 3: Three-dimensional μOCT video imaging captures neutrophil migration across human airway basal cell-derived epithelial layer towards exogenous chemoattractant, fMLP.

Video 4: Two-dimensional μOCT video imaging displays neutrophil migration across PAO1-infected human airway basal cell-derived epithelial layer.

Video 5: Human airway basal cell-derived epithelium was pre-treated with vehicle control (DMSO 1:1000) then washed prior to migration assay. Three-dimensional μOCT video imaging displays normal neutrophil migration across PAO1-infected human airway basal cell-derived epithelial layer following treatment with vehicle control.

Video 6: Human airway basal cell-derived epithelium was pre-treated with 12-lipoxygenase inhibitor, CDC (50μm) then washed prior to migration assay. Three-dimensional μOCT video imaging displays reduced neutrophil migration across PAO1-infected human airway basal cell-derived epithelial layer following treatment with CDC.

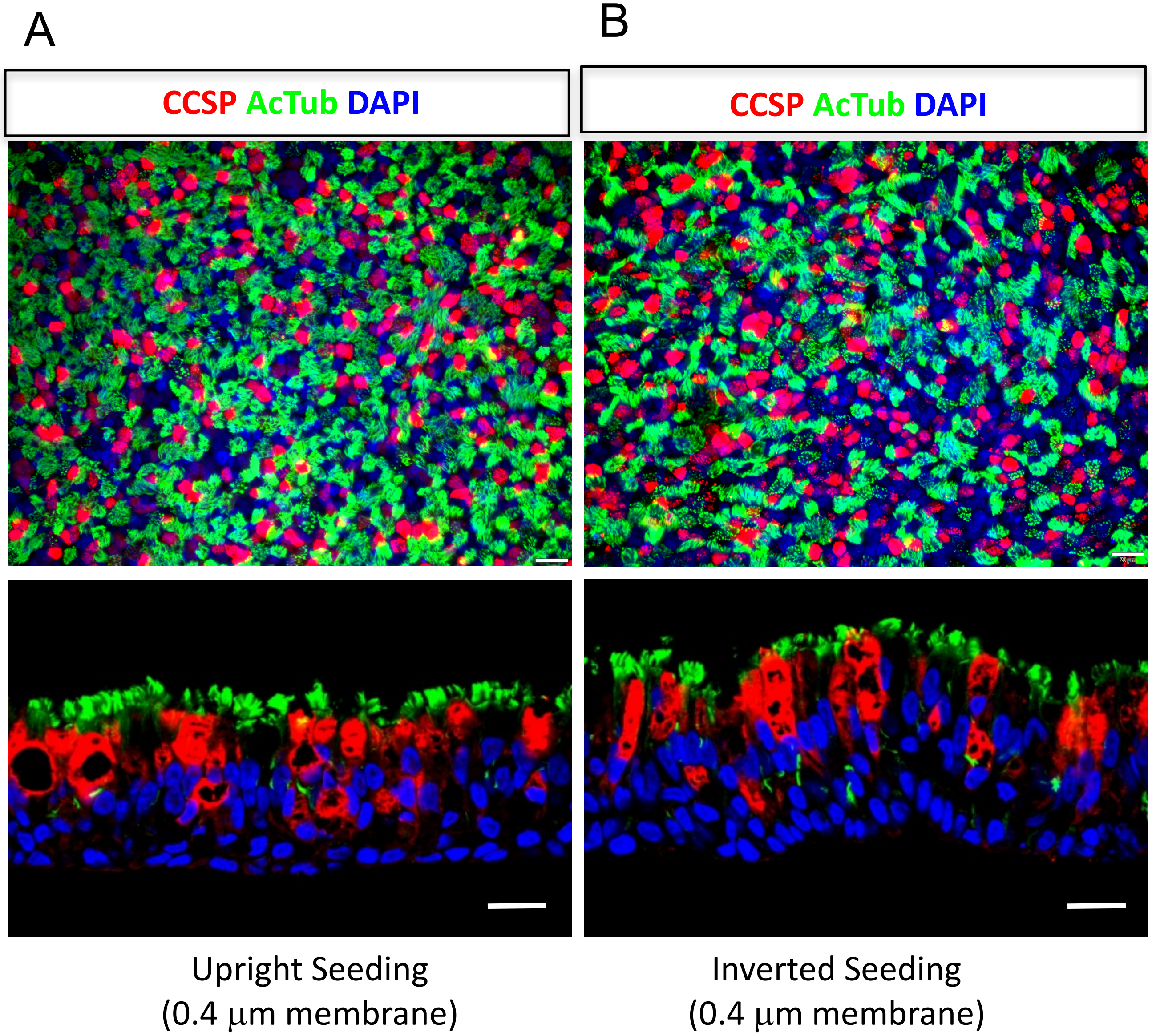

Supplemental Figure 1

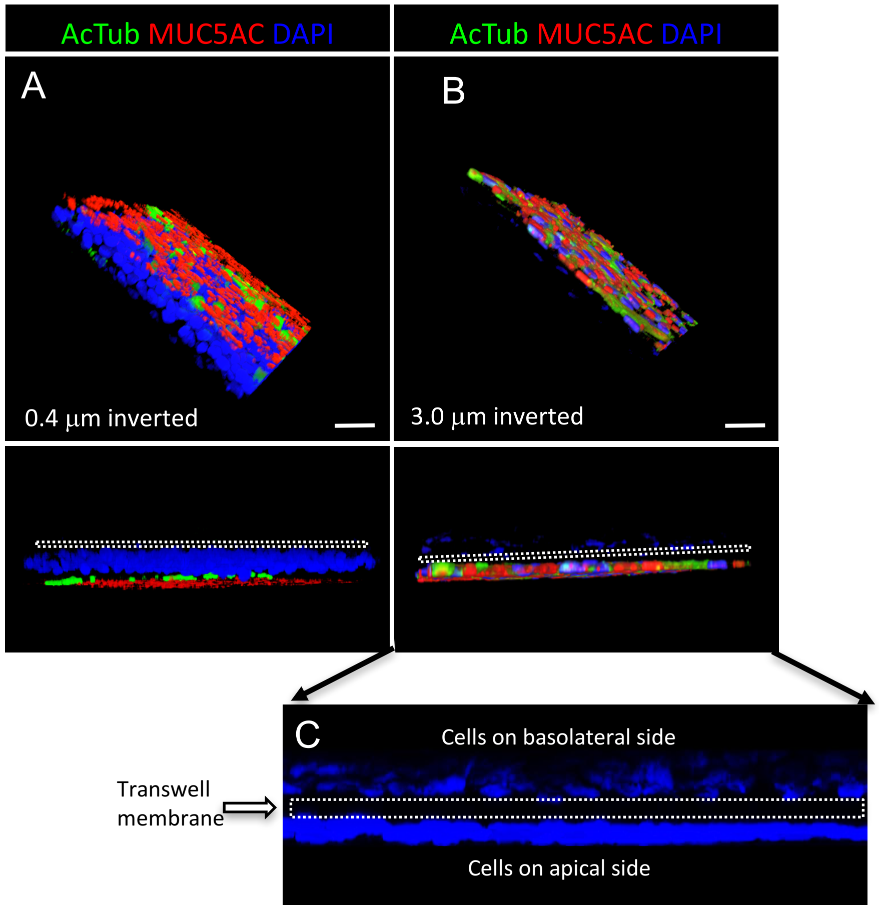

Supplemental Figure 2

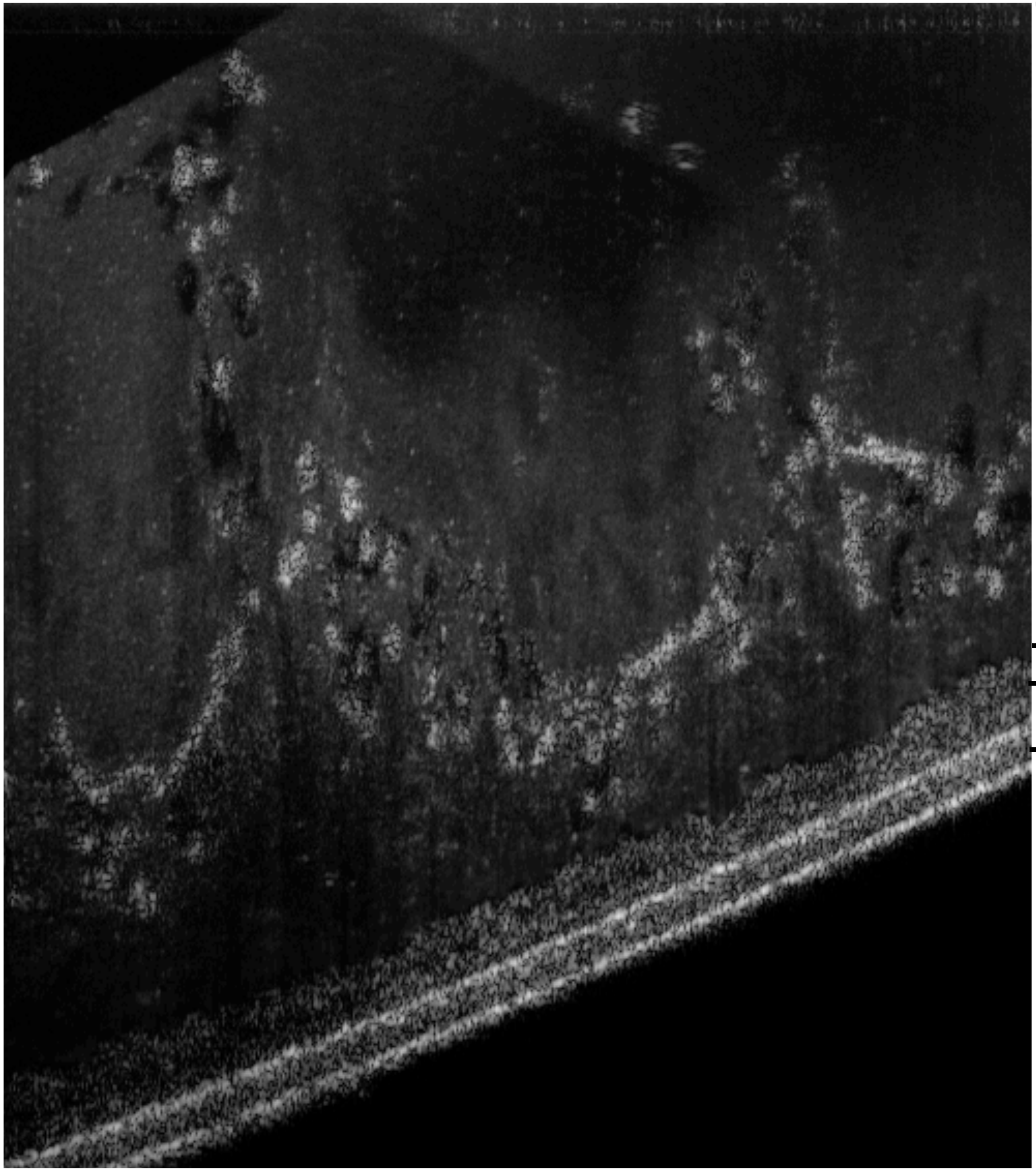

Supplemental Figure 3

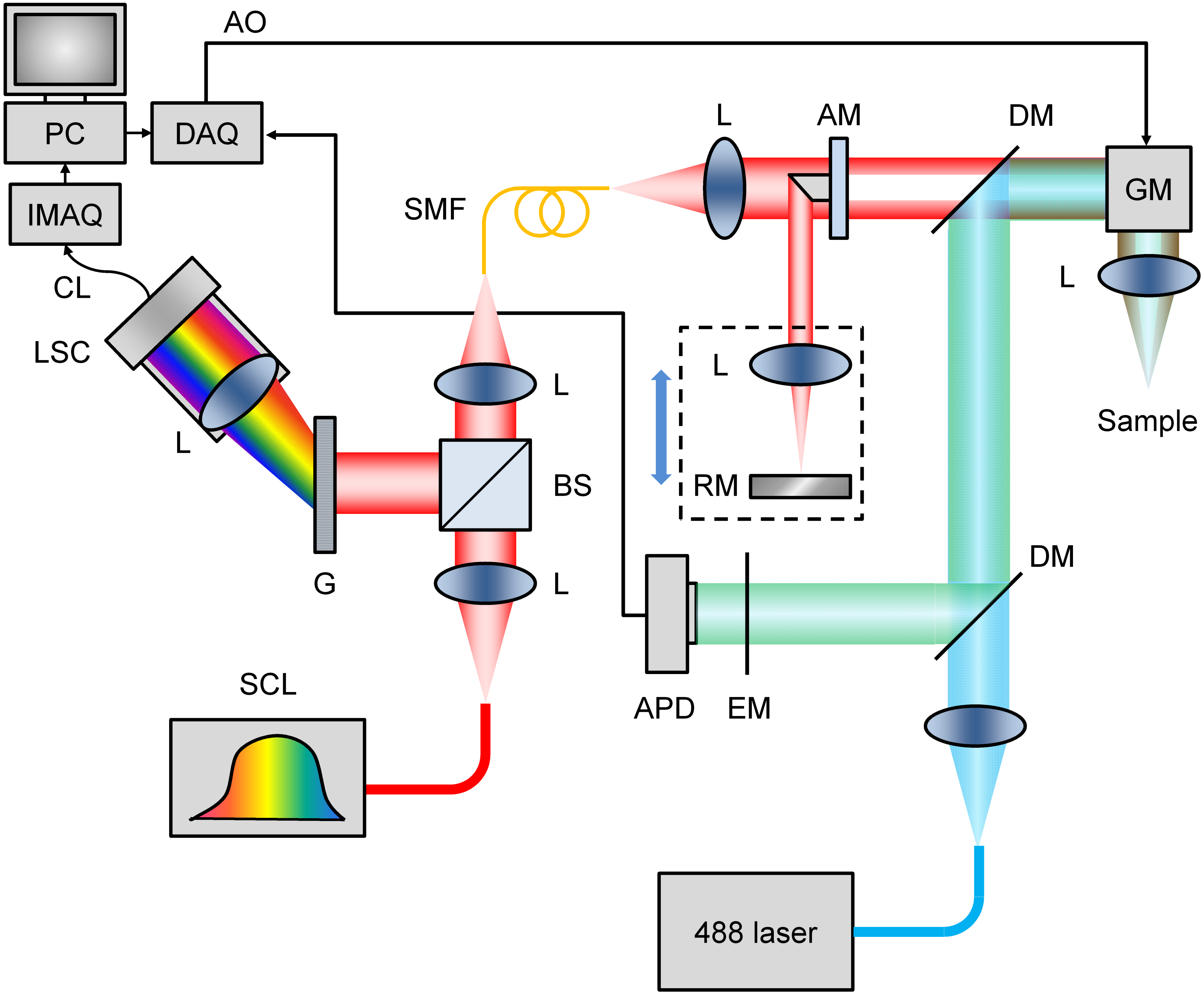

Supplemental Figure 4
